# Syphilitic Aortitis with Concomitant Neurosyphilis in Asymptomatic Patient

**DOI:** 10.3201/eid3107.250646

**Published:** 2025-07

**Authors:** Evan Czulada, Quinn Seau, Tyler Geshay, Danny Rayes, Trevor Wyand, Ethan Fraser, Ronald Beaulieu, Justin Beckett

**Affiliations:** Emory University Hospital, Atlanta, Georgia, USA (E. Czulada); Georgetown University School of Medicine, Washington, DC, USA (E. Czulada, Q. Seau); Georgetown University Hospital, Washington (T. Geshay, D. Rayes, T. Wyand, R. Beaulieu, J. Beckett); National Institutes of Health, Bethesda, Maryland, USA (E. Fraser).

**Keywords:** syphilis, aortitis, neurosyphilis, epidemiology, emerging disease, multimodality imaging, *Treponema pallidum*, bacteria, infection, Washington DC, United States

## Abstract

We report a rare case of syphilitic aortitis with possible neurosyphilis in an asymptomatic 89-year-old man in Washington, DC, USA. This case highlights the need to consider emerging infectious causes of aortitis, even in patients without classic risk factors, by using multimodality imaging with confirmatory serologic and cerebrospinal fluid testing.

Syphilitic aortitis is exceptionally rare. However, syphilis, caused by the bacterium *Treponema pallidum*, has reemerged as a global public health concern, and the Centers for Disease Control and Prevention has reported >200,000 cases in the United States in 2022 (*1*). After 10–30 years, ≈10% of untreated persons will develop syphilitic aortitis. Early recognition and treatment are imperative to avoid high rates of death (*2*), although the broad differential of aortitis can complicate diagnosis. In this article, we describe a rare case of syphilitic aortitis in an asymptomatic, elderly patient in which using imaging, serology, and epidemiologic context helped guide diagnosis.

An 89-year-old man with a history of chronic lymphedema, deep vein thrombosis, and peripheral artery disease sought care at Georgetown University Hospital in Washington, DC, USA, having experienced 1 week of right leg erythema and swelling. At admission, his laboratory results revealed leukocytosis and elevated inflammatory markers. Computed tomography (CT) of the patient’s right leg revealed soft tissue changes consistent with cellulitis unresponsive to antimicrobial drugs. Because of the concern for sepsis, we broadened the patient’s antimicrobial regimen, and an infectious disease consultation prompted further evaluation for alternative sources.

Contrast-enhanced CT of the chest showed circumferential soft tissue enhancement of the ascending aorta, aortic arch, and branching vessels, suggestive of aortitis versus intramural hematoma (Appendix Figure). Follow-up magnetic resonance angiography confirmed a diagnosis of aortitis (Figure). We consulted rheumatology because of the patient’s elevated inflammatory markers but had low suspicion for autoimmune disease after more specific antibody testing results were negative.

**Figure:**
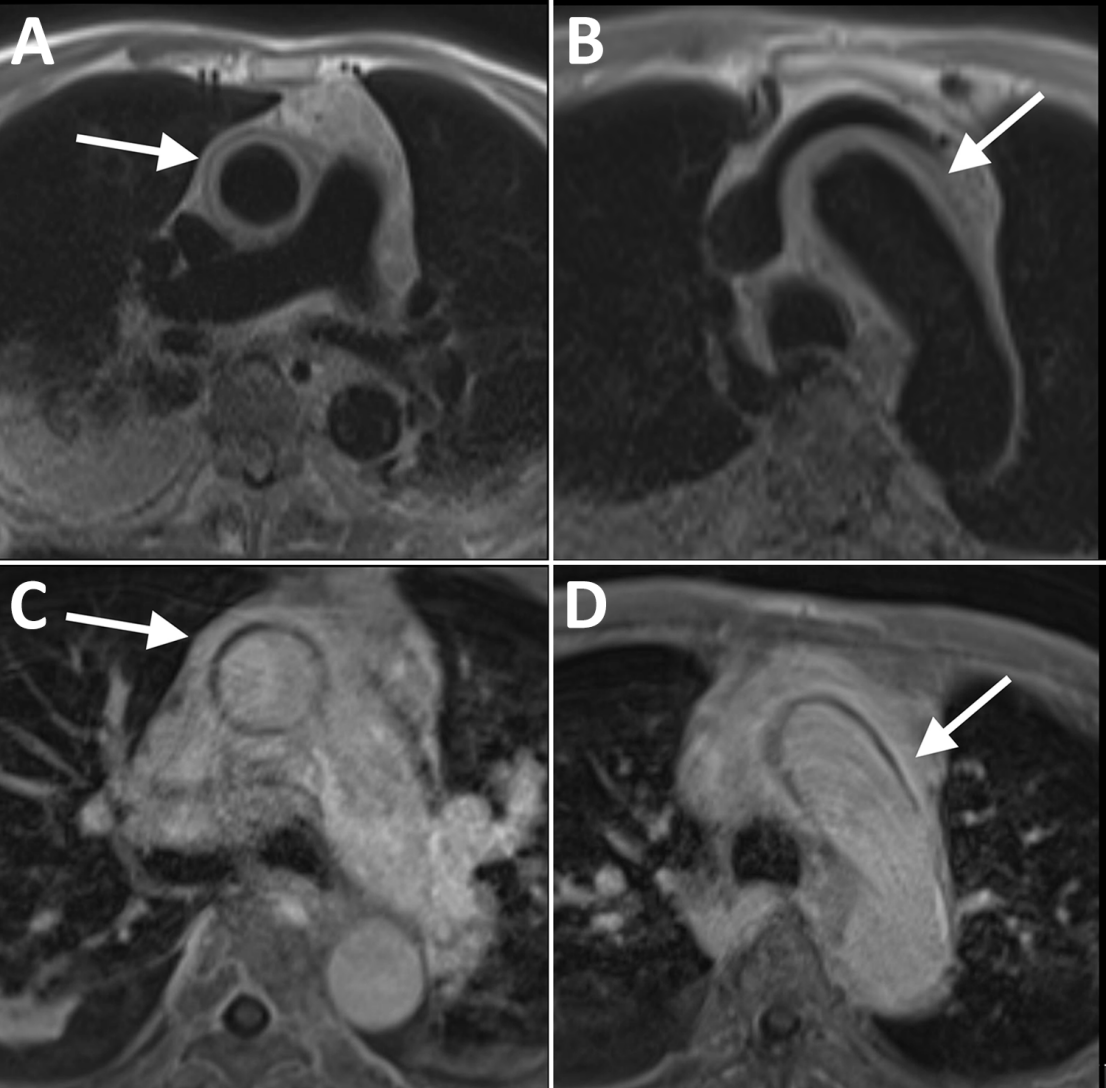
Electrocardiogram-gated magnetic resonance imaging of the ascending aorta in an 89-year-old patient with syphilitic aortitis and concomitant neurosyphilis, Washington, DC, USA. A, B) A circumferential periaortic T2 hyperintense signal was depicted at the main pulmonary artery (A) and aortic arch (B) on black blood prepared half-Fourier acquisition single-shot turbo spin-echo sequence images (white arrows). C, D) Contrast-enhanced T1-weighted magnetic resonance images at the main pulmonary artery (C) and aortic arch (D) show wall thickening and enhancement (white arrows) compatible with aortitis.

Because of our concern for an infectious etiology, we pursued serologic testing. Initial chemoluminescence immunoassay testing was reactive for syphilis, and subsequent rapid plasma reagin testing was positive with a 1:4 titer result. Despite no neurologic symptoms, we conducted cerebrospinal fluid (CSF) analysis that was negative by Venereal Disease Research Laboratory (VDRL) testing but positive for fluorescent treponemal antibody absorption. Full CSF results revealed a slightly elevated red blood cell count, slightly low monocytes, and unremarkable leukocyte and protein counts. Findings were inconsistent across all samples.

Ultimately, we attributed the patient’s underlying aortic pathology to syphilitic aortitis with concomitant neurosyphilis. The patient was treated with a 7-day course of intravenous penicillin G (3 million units every 4 h), and was prescribed the same treatment for an additional 7 days at discharge. The patient was discharged with reduced leg swelling, unremarkable vital signs, and resolved leukocytosis, but he was lost to follow-up.

This article describes the diagnosis of syphilitic aortitis with suspected neurosyphilis in an asymptomatic, elderly man with no classic symptoms or risk factors after using multimodality imaging and confirmatory testing. At 89 years of age, this patient is unusual and has a rare manifestation of syphilis, which is again rising in prevalence.

Our patient reported no cardiovascular symptoms, no prior syphilis diagnosis or treatment, no recollection of genital lesions, and no history of high-risk sexual behavior. Of note, he resided in Washington, DC, where syphilis rates are among the highest in the United States and have increased each year (*3*). Those data underscore the importance of considering *T. pallidum* infection in patients from high-risk areas, even in the absence of symptoms or traditional risk factors. Prompt treatment is critical, because some cases of syphilitic aneurysmal formation confer a 2-year mortality rate >80% (*4*). However, recognizing syphilitic aortitis can be challenging because of longstanding limitations in diagnostic strategies.

For source identification and subsequent control, whole-body imaging is typically indicated. Although CT imaging is sufficient for most aortopathies (*2*), it cannot reliably differentiate between hematoma and aortitis because of tissue density matching. Accordingly, a magnetic resonance aortogram provided further delineation of the abnormality, which was more indicative of aortitis than a hematoma because a hematoma is usually irregularly shaped and primarily diagnosed by using density-based imaging such as CT. Although not used in this case, fluorodeoxyglucose positron emission tomography/CT can be useful to evaluate differential causes of unexplained fever associated with vasculitis and ascertain treatment efficacy in cases of tertiary syphilis (*5*–*7*).

After ruling out more common autoimmune diseases, we chose treponemal-specific chemoluminescence immunoassay testing for suspicion of syphilitic aortitis. We selected this reverse sequence strategy because of institutional preference and high pretest probability (*8*), and a positive rapid plasma reagin titer of 1:4 confirmed active syphilis. Furthermore, diffuse syphilitic disease of the aorta necessitated CSF testing (*9*), showing negative VDRL and positive fluorescent treponemal antibody absorption. VDRL testing has poor sensitivity, and fluorescent treponemal antibody absorption has poor specificity. Fluorescent treponemal antibody absorption might be influenced by trace red blood cell contamination from the CSF (*10*). Nonetheless, intravenous penicillin G was initiated because of patient preference and the risk of not treating our elderly patient.

Syphilitic aortitis remains a rare but challenging diagnosis, especially in patients without typical symptoms or risk factors. This report highlights the importance of stepwise imaging and serologic testing in identifying syphilitic aortitis. CT scan is a reasonable first radiographic study, but magnetic resonance imaging can better define the underlying etiology. As syphilis cases continue to rise, clinicians should use local epidemiologic trends to ensure early disease detection.

Appendix Additional information about syphilitic aortitis with concomitant neurosyphilis in asymptomatic patient.
