# Supplementary material for: Syphilitic Aortitis with Concomitant Neurosyphilis in Asymptomatic Patient
Source: Emerg Infect Dis. 2025 Jul;31(7):1486–9. doi: 10.3201/eid3107.250646 (PMC12205471; doi:10.3201/eid3107.250646)
Supplement: Appendix — Additional information about syphilitic aortitis with concomitant neurosyphilis in asymptomatic patient. [file 25-0646-Techapp-s1.pdf]

Article DOI: https://doi.org/10.3201/eid3107.250646

EID cannot ensure accessibility for supplementary materials supplied by authors. Readers who have difficulty accessing supplementary content should contact the authors for assistance.

Syphilitic Aortitis with Concomitant Neurosyphilis in Asymptomatic Patient

Appendix

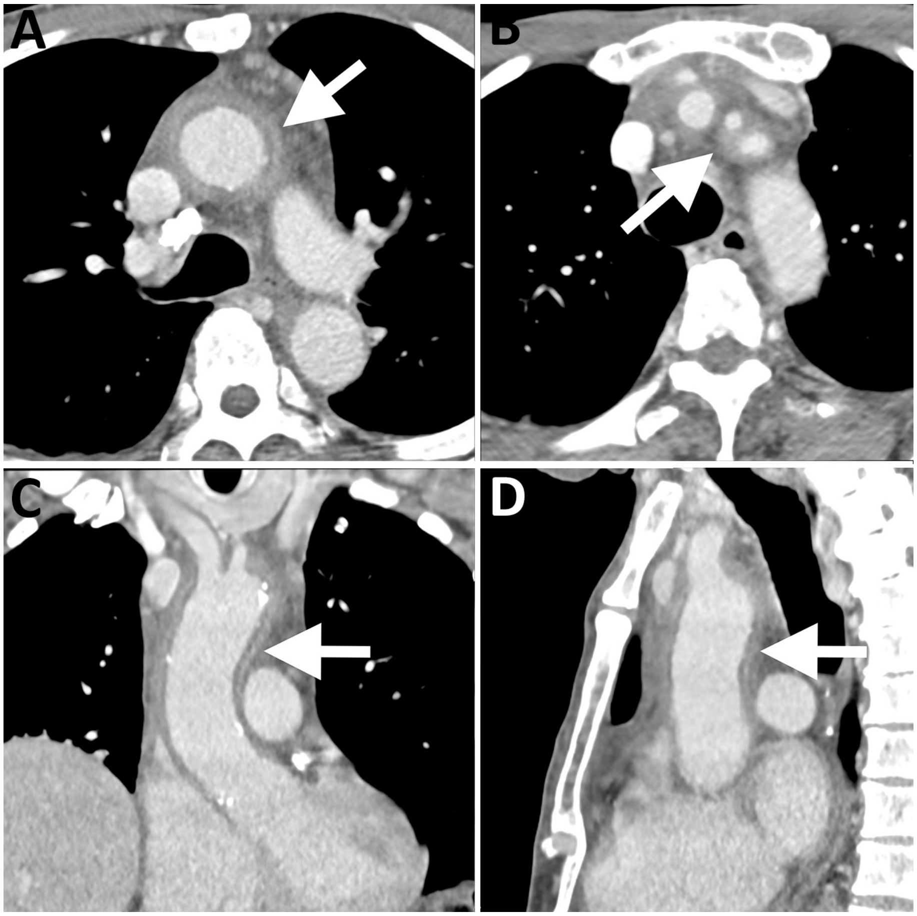

Appendix Figure. Contrast-enhanced computed tomography of the ascending aorta in an 89-year-old patient with syphilitic aortitis and concomitant neurosyphilis. Axial images at the level of the A) sinotublar junction and B) the aortic arch. White arrows indicate a diffuse circumferential enhancing soft tissue rim

around the aorta. C) Coronal and D) sagittal images demonstrate extent of involvement. White arrows indicate diffuse involvement from the aortic sinotubular junction to the proximal aspect of the arch vessels.
